# In Silico Studies of Four Compounds of *Cecropia obtusifolia* against Malaria Parasite

**DOI:** 10.3390/molecules28196912

**Published:** 2023-10-03

**Authors:** Carlos Alberto Lobato-Tapia, Yolotl Moreno-Hernández, Zendy Evelyn Olivo-Vidal

**Affiliations:** 1 Departamento de Ingeniería en Biotecnología, Universidad Politécnica Metropolitana de Puebla, Popocatépetl s/n, Reserva Territorial Atlixcáyotl, Tres Cerritos, Puebla 72480, Mexico; 2 Departamento de Salud, El Colegio de la Frontera Sur Unidad Villahermosa, Carretrea Federal Villa-Hermosa-Reforma Km 15.5, Ra. Guineo Segunda Sección, C.P., Villahermosa 86280, Mexico; yolotl.moreno@estudianteposgrado.ecosur.mx

**Keywords:** antimalarial, plasmodium, molecular docking, molecular dynamics, *Cecropia obtusifolia*, pharmacokinetics

## Abstract

Malaria is a disease that affects many people in the world. In Mexico, malaria remains an active disease in certain regions, particularly in the states of Chiapas and Chihuahua. While antimalarial effects have been attributed to some species of Cecropia in various countries, no such studies have been conducted in Mexico. Therefore, the objective of this study was to evaluate the in silico antimalarial activity of some active compounds identified according to the literature in the species of *Cecropia obtusifolia*, belonging to the *Cecropiaceae* family, such as ursolic acid, α-amyrin, chrysin, and isoorientin. These compounds were evaluated with specific molecular docking and molecular dynamics (MD) studies using three different malarial targets with the PDB codes 1CET, 2BL9, and 4ZL4 as well as the prediction of their pharmacokinetic (Pk) properties. Docking analysis revealed the following best binding energies (kcal/mol): isoorientin–1CET (−9.1), isoorientin–2BL9 (−8.8), and chrysin–4ZL4 (−9.6). MD simulation validated the stability of the complexes. Pharmacokinetics analysis suggested that the compounds would generally perform well if administered. Therefore, these results suggest that these compounds may be used as potential drugs for the treatment of malaria.

## 1. Introduction

Malaria is a disease that affects many people worldwide but mainly newborns, children under five years of age, pregnant women, travelers, and people with a compromised immune system are at risk of developing a major infection [1]. It is a disease caused by the bite of a female mosquito of the genus *Anopheles* spp. which requires feeding for reproduction; the infection of this disease causes symptoms such as fevers, headache, and chills, symptoms that can be implicated in fatigue, confusion, seizures, shortness of breath, anemia, coma, and death [1,2].

In 2021, half of the world’s population was at risk of malaria and during the same year, there were 247 million cases and 619,000 deaths mainly in the African region where the incidence is 232.8 (cases per 1000 people at risk) [2]. *P. vivax* is the second most studied species, only below *P. falciparum* due to the severity of the disease it causes and its wide distribution; in Central America alone, *P. vivax* is the etiologic agent of 95% of diagnosed malaria cases and in the region of the Americas the incidence is 4.6. Some standardized treatments to combat this disease have been generating resistance due to bad practice or continuous use. Many of the current drugs have been developed by the use of plants in a traditional way and by studies of some compounds present in the species with some activity, such as some flavonoids of plant species that have been described with antimalarial potential, although many more are unknown [3,4,5]. In Mexico, malaria remains endemic in states like Chiapas and Chihuahua where several communities are affected year after year [6]. *Cecropia obtusifolia* is a perennial tree distributed from Mexico to Brazil, that has been extensively studied for its pharmacological properties and use in the treatment of chronic diseases [7,8,9]. These properties have been attributed to the presence of active compounds such as flavonoids, proantho-cyanins, terpenoids, steroids, chlorogenic acid, caffeic acid, storminous acid, and isoorientin [10,11,12], among others, primarily found in the leaves, bark, stem, flower, bud, and root of these plants [13]. Recently, this species has been attributed to moderate antiplasmodial effects, which could promote its use for the treatment of malaria [14], an infectious disease caused by the *Plasmodium* spp. parasite and transmitted to humans by the *Anopheles* spp. vector.

To date, no studies have evaluated the potential antimalarial activity of some compounds of *Cecropia obtusifolia*. On the other hand, some of the most targeted molecular or therapeutic sites in the search for antimalarial drugs are the proteins 2BL9, 4ZL4, and 1CET. The protein 2BL9, or dihydrofolate reductase protein of *Plasmodium vivax*, is a crucial target in the development of new antimalarial agents [15,16] such as pyrimentamine, a treatment for the elimination of protozoa like plasmodium or toxoplasmosis [17]. Similarly, the protein 4ZL4 belongs to the aspartyl protease known as plasmepsin V of *P. vivax* and it is the site of action of synthetically formulated compounds to combat malaria such as WEHI-842 [12,18,19,20]. Lastly, the protein 1CET is associated with the *Plasmodium falciparum* lactate dehydrogenase cofactor binding site, which is the main binding and action site for chloroquine, one of the most widely used drugs to treat malaria [19,21,22].

Therefore, the main objective of this study was to evaluate the potential antimalarial activity of the metabolites identified formerly in *C. obtusifolia* through in silico analysis, as well as to assess their pharmacokinetic properties.

## 2. Results

### 2.1. Compounds of Cecropia obtusifolia

**Figure 1:**
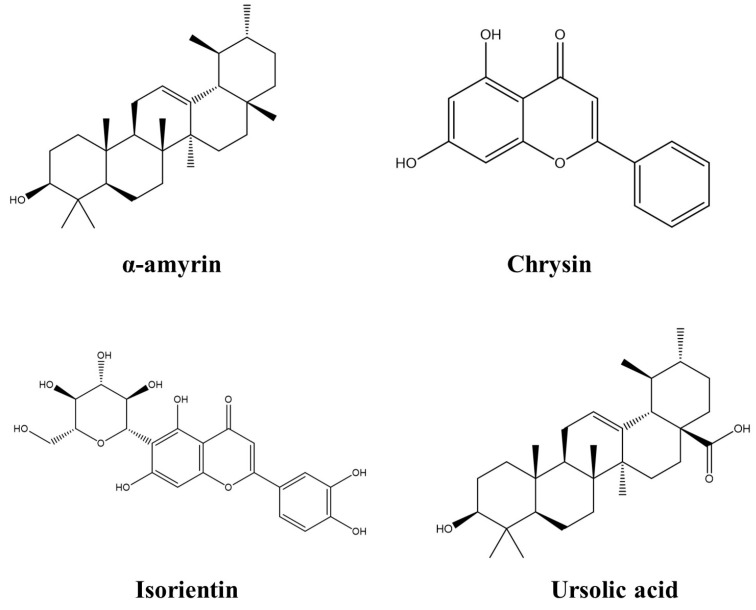
Chemical structures of the compounds identified in *C. obtusifolia* and evaluated against target proteins in malaria.

Due to some background in the use of flavonoids from other species with potential antimalarial activity, it was decided to work with flavonoids such as chrysin, isoorientin, and triterpenes such as ursolic acid and α-amyrin present in this species Figure 1.

### 2.2. Molecular Docking

Molecular docking analysis was performed to assess the interaction capacity between the target proteins and the compounds found in *C. obtusifolia*, using Autodock Vina to calculate the binding energy. The three target proteins used were 2BL9, 4ZL4, and 1CET, which play a key role in malaria treatment and have known binding sites in their crystalline structures (found in the .pdb file) with a 25 Å grid.

The binding energy of all compounds was below −7.7 kcal/mol, as shown in Table 1. The best complexes between the ligands and target were chrysin–2BL9 (−8.7 kcal/mol), chrysin–4ZL4 (−9.6 kcal/mol) and isoorientin–1CET (−9.1 kcal/mol). In fact, almost all the evaluated ligands exhibited higher affinities than the co-crystallized ligands in the re-docking, except for ursolic acid with 4ZL4 (−7.8 kcal/mol) compared to Wehi (−8.2 kcal/mol).

**Figure 2:**
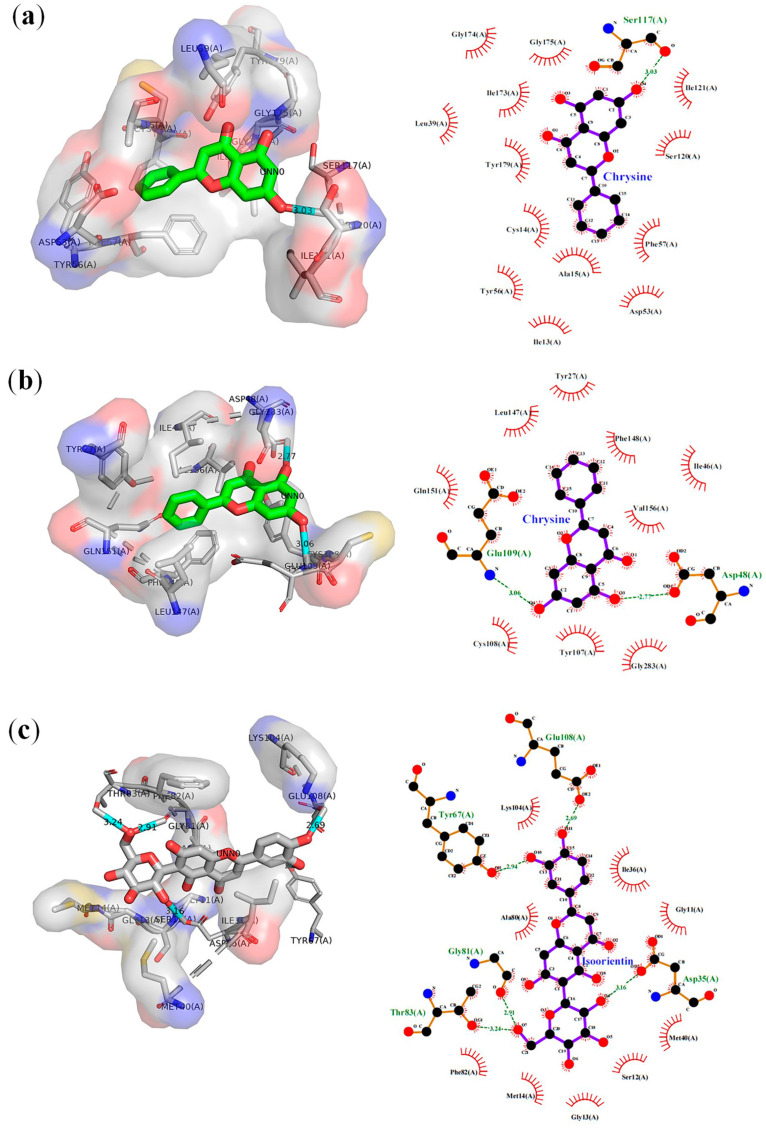
Two- and three-dimensional representations of the hydrogen bonding and hydrophobic interaction between ligands within the binding cavity of receptors. (**a**) Chrysin–2BL9 complex, (**b**) chrysin–4ZL4 complex, and (**c**) isoorientin–CET complex.

Figure 2 illustrates the binding site and orientation pose of these three complexes. The interactions formed between these compounds and the target proteins were visualized using PyMOL 2.5.4 and LigPlot 2.2.5. Chrysin was found to form hydrophobic interactions with Ile13, Cys14, Ala15, Asp53, Tyr56, Phe57, Ser120, Ile121, Ile173, Gly174, and Gly175 and hydrogen bond with Ser117 (3.03 Å) from the 2BL9 target. Chrysin also exhibited hydrophobic interactions with Tyr27, Ile46, Tyr107, Cys108, Leu147, Phe148, Gln151, Val156, and Gly283, along with two hydrogen bonds with Asp48 (2.77 Å) and Glu109 (3.06 Å) from 4ZL4. Isoorientin demonstrated hydrophobic interactions with Gly11, Ser12, Gly13, Met14, Ile36, Met40, Ala80, Phe82, and Lys104 as well as five hydrogen bonds with Asp35 (3.16 Å), Gly81 (2.91 Å), Thr83 (3.24 Å), and Glu108 (2.69 Å) from 1CET.

Overall, chrysin showed the strongest binding affinity for both 2BL9 and 4ZL4. Its interaction with 2BL9 involved residues Cys14, Ala15, Asp53, Phe57, Ile173, and Tyr179 which coincide with pyrimethamine. In contrast, its interaction with 4ZL4 only aligned with a Wehi-binding residue (Asp48). The high affinity of chrysin for these two receptors positions it as a promising compound for malaria treatment, particularly in the eradication of *P. vivax*. On the other hand, isoorientin demonstrated the highest affinity among the compounds tested with 1CET.

The validation of molecular docking was performed by re-docking the ligands into the active target sites. Ligand re-docking was conducted after randomizing the ligand’s conformation and orientation. RMSD values were manually calculated using PyMOL, resulting in the following values: 0.635 Å (1CET-Chloroquine), 0.333 Å (4ZL4-WEHI), and 0.639 Å (2BL9-pyrimethamine).

### 2.3. Molecular Dynamics

After evaluating the protein–ligand interactions and obtaining satisfactory results, molecular dynamics (MD) analysis was conducted on the ligands with the highest affinity energies to their targets to assess the stability of the protein–ligand complexes. MD simulations were performed for 50 ns for the complexes with the lowest affinity energy: chrysin–2BL9, chrysin–4ZL4, and isoorientin–1CET.

To analyze the structural stability and fluctuations of the complexes, the trajectories were analyzed using RMSD (root mean square deviation), RMSF (root mean square fluctuation), RG (radius of gyration), and SASA (solvent accessible surface area).

**Figure 3:**
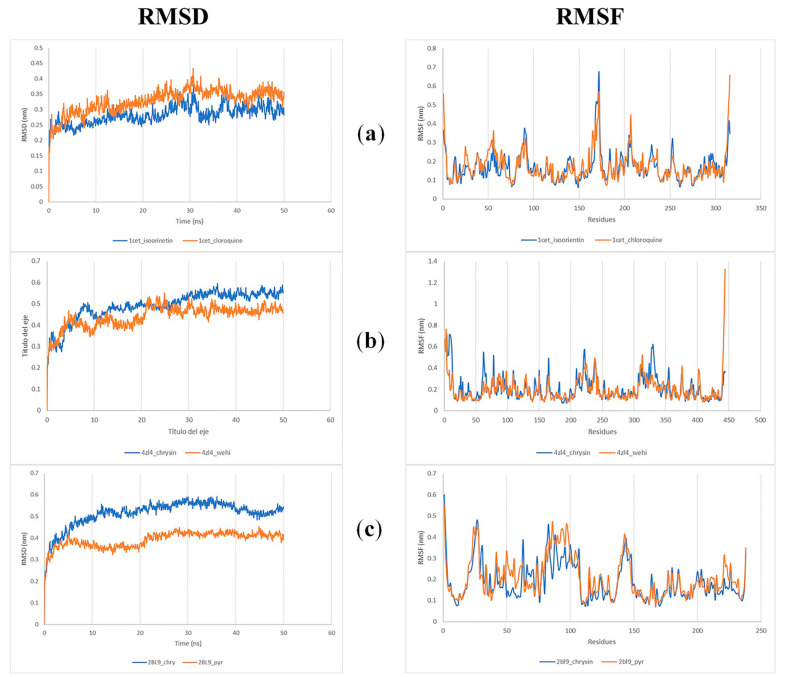
MSD and RMSF plots for 50 ns MD simulation of 1CET–isoorietin (**a**), 4ZL4–chrysin (**b**), and 2BL9–chrysin (**c**) complex versus co-crystallized ligand.

The RMSD of the protein backbone was plotted against the 50 ns simulation duration to assess variations in structural conformation (Figure 3). The chrysin–2BL9 complex exhibited significant variation (0.3 nm) around 20 ns, but after this time, it achieved a stable conformation without significant deviations in the RMSD values. The chrysin–4ZL4 complex showed variations in backbone RMSD ranging between 0.4 to 0.7 nm from 10 to 30 ns. The stable conformation was reached between 33 and 50 ns with no considerable deviations in the values. The isoorientin–1CET complex reached equilibrium after 15 ns and remained stable until 50 ns. The RMSD values for both the 4ZL4–chrysin and 2BL9–chrysin complexes were slightly higher than those of the proteins bound to their co-crystallized ligands, while the 1CET–isoorientin complex showed lower values compared to the control.

The RMSF plot was used to analyze the flexibility of each amino acid residue in the target protein upon binding to a ligand (Figure 3). In the chrysin–2BL9 complex, the amino acids that interacted with chrysin showed minimal fluctuation values. Although some residues (30, 31, 63, 74, 81–84, 96, and 141–144) displayed larger fluctuations, these residues are not related to the binding site pocket. However, in the chrysin–4ZL4 complex, a greater number and larger fluctuations of residues were observed throughout the protein. The residues interacting with the ligand did not show high fluctuations but greater variability was observed in other regions. Finally, the isoorientin–CET complex exhibited minimal fluctuation values, except for residues 169–172, which are not involved in the binding site pocket. The fluctuations observed with the evaluated compounds are very similar to those of the co-crystallized complexes.

**Figure 4:**
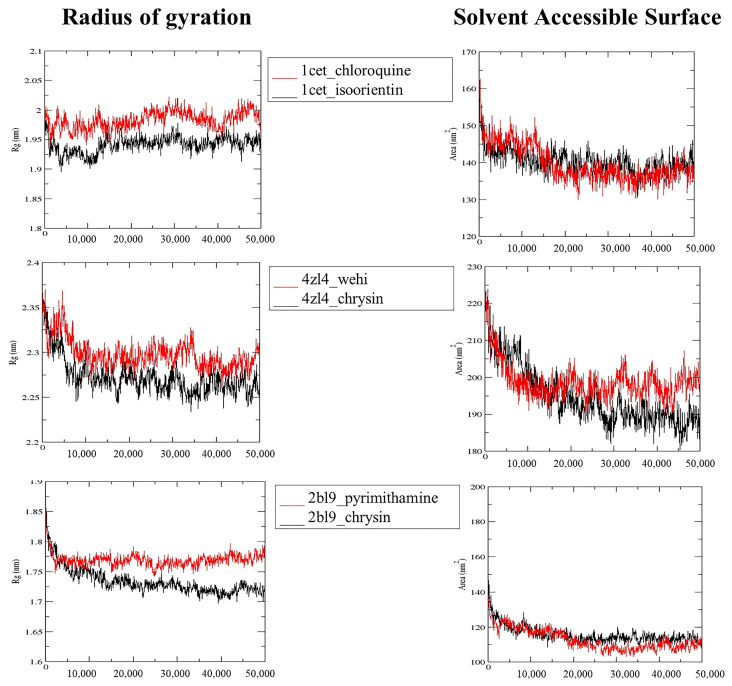
Radius of gyration and solvent accessible surface area plots for 50 ns MD simulation of the 1CET–isoorietin, 4ZL4–chrysin, and 2BL9–chrysin complex versus co-crystallized ligand.

RG values of the protein backbone atoms were used to analyze the compactness and structural flexibility of the complex over simulation time (Figure 4). In the chrysin–2BL9 complex, the RG values decreased until 10 ns and remained relatively stable thereafter. Similarly, in the chrysin–4ZL4 complex, the RG values decreased after 10 ns with small fluctuations, albeit greater than in the chrysin–2BL9 complex. However, in the isoorientin–1CET complex, an increase in RG value was observed after approximately 15 ns, remaining stable until the end of the dynamics. When comparing these results with those obtained from the co-crystallized ligands, it is evident that the compounds from *C. obtusifolia* tend to have lower RG values.

Furthermore, the changes in the surface area (SASA) for all complexes were plotted against time to estimate the variations (Figure 4). In all complexes, a decrease in SASA values was observed until 20 ns. Moreover, both the 1CET–isoorientin and 4ZL4–chrysin complexes exhibited a greater decrease in SASA compared to when the targets were bound to their co-crystallized ligands (chloroquine and Wehi, respectively). In contrast, the 2BL9–chrysin complex showed average SASA values higher than those with the co-crystallized ligand (pyrimithamine).

### 2.4. Pharmacokinetic Properties

The ADMET properties of four compounds found in *C. obtusifolia* were assessed using the ADMETlab 2.0 web server. The main pharmacokinetics parameters are presented in Table 2 and complete results can be reviewed in the Supplementary Material Table S1. Isoorientin was the only compound that did not meet the criteria according to Lipinski’s five rules and was not considered a potential drug. Similarly, isoorientin exhibited a low LogP value, indicating higher water solubility compared to the other compounds which are more liposoluble. The results also showed that α-amyrin, chrysin, and ursolic acid have the potential for excellent intestinal absorption (HIA) and permeability through Caco-2 cells, as indicated by ADMETlab 2.0 interpretation ranges. However, isoorientin is expected to have poor absorption and permeability.

The distribution parameter was evaluated for plasma protein binding (PPB), volume distribution (VD), and the blood–brain barrier (BBB). All the compounds exhibited very high affinity to plasma proteins (PPB values greater than 90%), resulting in a low therapeutic index. However, the results obtained for VD and BBB were appropriate for all tested compounds. In terms of excretion, isoorientin and ursolic acid showed low clearance (CL), while α-amyrin and chrysin exhibited good clearance. The half-life (T1/2) was long for α-amyrin and ursolic acid but short for chrysin and isoorientin.

Regarding toxicity, none of the compounds were found to be probable inhibitors of the human ether-a-go-go-related gene (hERG) channels or carcinogenic. However, only ursolic acid presented an intermediate probability of being hepatotoxic to humans (H-HT).

## 3. Discussion

Malaria is an infectious disease caused by the protozoa parasite *Plasmodium* spp. and transmitted by mosquitoes of the *Anopheles* spp. genus [23]. Despite numerous efforts, the World Health Organization (WHO) acknowledges that insufficient progress has been made towards its eradication [24]. Therefore, it is crucial to continue the search for new compounds that exhibit enhanced activity against the parasite while minimizing side effects [25]. Plants remain an important source for discovering biologically active compounds of interest [26,27,28]. Hence, in this study, we focused on identifying and evaluating the antimalarial activity of specific compounds present in *C. obtusifolia* compounds such as α-amyrin, ursolic acid, chrysin, and isoorientin; the degree of purity of a compound, identifying and comparing analytes, and monitoring a reaction. It is easy to understand and execute, allowing separations in a short time and them to be versatile and low cost. In addition, only small amounts of reagents, standards, and samples are used for the identification of different compounds even in the same run [29,30]. These compounds have been previously reported in research involving species belonging to the Cecropeacea family, as demonstrated by studies such as Costa et al., 2011 [13], Rivera-Mondragon et al., 2017 [14], and others.

To evaluate the antimalarial activity, we utilized computer-aided drug design (CADD) tools. CADD encompasses various techniques such as de novo drug design, receptor-based ab initio pharmacophore modeling, dynamic trajectory water pharmacophores, free energy perturbation calculations, polypharmacology, and big data analysis [31,32,33]. One of these tools is molecular docking, which allows for the assessment of binding affinity between a target protein and a chemical compound of interest, thereby predicting the compound’s ability to bind effectively to the protein [34,35,36]. In our study, we evaluated the binding affinity between the four compounds identified in *C. obtusifolia* and three pharmacologically relevant proteins from *Plasmodium* spp.: dihydrofolate reductase protein (2BL9) [17], aspartic protease (4ZL4) known as Plasmepsin V, both from *P. vivax* [18], and lactate dehydrogenase (1CET) from *P. falciparum* [21].

Regarding the targets of interest, dihydrofolate reductase (2BL9) is a target of interest for the treatment of various infectious diseases, including leishmaniasis, tuberculosis, bacterial infections, and malaria, among others [37]. It plays a crucial role alongside thymidylate synthase in the recycling of folates, which is important for the synthesis of purines and methionine [38]. Upon infecting erythrocytes, *Plasmodium* spp. releases numerous proteins into the cytosol that are essential for its survival in this environment. One such vital enzyme in these pathways is the aspartic protease plasmepsin V (4ZL4) [39]. Additionally, this enzyme plays a significant role during gametocytogenesis. On another note, lactate dehydrogenase (1CET) functions primarily in the conversion of pyruvate to lactate, a crucial process for energy production in most living organisms. In the asexual stages of *P. falciparum*, lactate dehydrogenase produces lactate as a byproduct associated with growth regulation [40]. This enzyme is considered an important drug target in malaria treatment and inhibiting its activity leads to the parasite’s demise.

Having compounds that can inhibit this type of enzyme is a key aspect of the development of new antimalarial drugs [41,42,43]. Therefore, in this study, we evaluated the ability of *C. obtusifolia* compounds to bind to and inhibit these enzymes and determined their potential as antimalarial agents. Our compounds exhibited greater binding affinity compared to the control compounds obtained from the provided .pdb file, except for the 4ZL4-ursolic acid complex. Among the compounds tested, chrysin demonstrated the highest affinity for both 2BL9 (−8.7 kcal/mol) and 4ZL4 (−9.6 kcal/mol); similar results to those obtained by Sivaramakrishnan et al. [44] against Plasmepsin V and Chaniad et al. [45] against *P. falciparum* lactate dehydrogenase. Chrysin is a natural polyphenol with documented biological activities, including anti-cancer and hepatoprotective properties [46]. Likewise, it has been verified that in vitro it inhibits the enzymes Falcipain-2 and Plasmepsin II [47] to an extent between 24 and 35%; it also showed activity in vitro assays against strains of *P. falciparum* sensitive and resistant to chloroquine [48]. On the other hand, isoorientin is a C-glucosyl flavone with verified biological activities, such as reducing hyperglycemia, hyperlipidemia, insulin resistance [49,50], anti-inflammatory effects [51], and activity against certain types of cancer [52,53]. Its antimalarial potential has also been evaluated [54], although its specific mechanism of action remains undetermined. Based on these findings, we propose that chrysin and isoorientin could be effective antimalarial drugs as they bind effectively to both *P. vivax* and *P. falciparum* receptors, exhibiting lower binding energies than the control compounds.

To assess the stability of the evaluated complexes from molecular docking, a molecular dynamics (MD) analysis was conducted on the best docking results. MD simulations investigate the dynamic behavior of proteins and their interactions with small molecules, such as drugs [55]. To confirm the stability of the analyzed complexes, several parameters including RMSD, RMSF, RG, and SASA were calculated.

RMSD, or root mean square displacement, measures the average deviation of backbone atoms in the protein–ligand complex from a reference structure (Rref) and is plotted over the simulation time. RMSF, or root mean square fluctuations, quantify the positional deviations of individual particles (atoms) in relation to their reference positions. The radius of gyration (RG) provides information about the overall size and structural changes of the protein during MD simulations [44]. The solvent accessible surface area (SASA) is a significant criterion that indicates the extent of receptor exposure to surrounding solvent molecules during the simulation [56].

By analyzing these parameters, we confirmed that chrysin and isoorientin do not significantly impact the stability of the studied targets. They remain attached to the proteins without causing structural disturbances, suggesting their potential to function effectively when administered and bound to specific protozoan proteins.

In order for a substance to be used as a medicine, it must undergo evaluations to determine its ability to efficiently navigate through the body, thereby producing the expected effects. Additionally, the substance’s potential toxicity needs to be evaluated. These assessments fall under the domain of ADMET, which encompasses pharmacokinetics [57]. Conducting such evaluations can be expensive and time-consuming; however, there are currently cheminformatics tools available that enable the prediction of chemical substance behavior within an organism [58]. In our study, we utilized the ADMETlab 2.0 platform to predict the pharmacokinetic parameters of the four compounds identified in *C. obtusifolia*. ADMETlab 2.0 is a web server designed for predicting the pharmacokinetics and toxicity properties of chemicals, including various endpoints such as physicochemical properties, medicinal chemistry properties, ADME properties, toxicity endpoints, and toxicophore rules [59].

Among the four compounds, isoorientin is the only one that may not be suitable for oral administration as it exhibits low intestinal absorption and poor permeability through Caco-2 cells. However, the possibility of administering it via non-oral routes is not ruled out. Distribution analysis indicates favorable volume distribution (VD) and high affinity to plasma protein binding (PPB) for all compounds, as well as the ability to penetrate the blood–brain barrier (BBB). This last aspect is particularly relevant for cases of cerebral malaria, as certain drugs may struggle to reach the brain due to this biological barrier [60,61].

Regarding excretion, α-amyrin demonstrates the most favorable values for both clearance and half-life (T1/2). However, the excretion values of the other compounds do not impose limitations on their potential administration; rather, dosage adjustments may be necessary based on their respective rates of excretion [62,63].

Toxicity is a critical consideration when assessing compounds for potential drug use in vivo. Therefore, it is crucial to determine any potential toxicity before initiating further analyses [64]. One of the primary concerns in this type of assessment is the inhibition of hERG channels which can lead to irregular heart rhythm and even cardiovascular arrest [65]. Fortunately, based on our results, none of the compounds exhibit hERG channel inhibition nor do they display carcinogenic potential. However, it should be noted that ursolic acid shows an intermediate probability of being hepatotoxic to humans.

## 4. Materials and Methods

### 4.1. Proteins Preparation

Crystal structures of key receptors for *P. vivax* (2BL9, 4ZL4) and *P. falciparum* (1CET) were obtained from the Protein Data Bank (http://www.rcsb.org/, accessed on 25 November 2022) with resolutions of 1.90 Å, 2.37 Å, and 2.05 Å, respectively. The structures were processed using the Dock Prep Module of UCSF Chimera 1.14 [66]. This involved removing water molecules, additional chains, and ligands, as well as adding hydrogen atoms and assigning partial charges. Any missing protein fragments were reconstructed with the assistance of I-TASSER [67].

### 4.2. Compound Retrieval and Preparation

The ligands (ursolic acid, α-amyrin, chrysin, and isoorientin) and control compounds (chloroquine, pyrimethamine, and WEHI-842) were obtained in a Mole2 file format from PubChem (https://pubchem.ncbi.nlm.nih.gov/, accessed on 25 November 2022). Avogadro 1.2.0 [68] was used for geometry optimization of the ligands and to convert the file format to .pdb. The prepared ligands were then used with the Chimera docking tool.

### 4.3. Molecular Docking Analysis

All structures were aligned and a large grid box accommodating all experimental ligands was used. A grid box of 25 × 25 × 25 Å was employed for all structures. The coordinates of the grid box for each target were as follows: 2BL9 (64.00, 70.00, and 60.38), 4ZL4 (70.99, 71.13, and 70.93), and 1CET (60.903, 75.211, and 50.674), based on the previous position of the natural ligand and providing additional space. The Lamarckian genetic algorithm (LGA) was performed with the rigid receptor molecule to search for the best conformers. Ten independent docking experiments were carried out for each structure using the Autodock Vina standalone tool [69,70]. Among the ten replicates, the most frequently recurring energy and pose were selected as the final result, which were visualized using LigPlot+ [71] and PyMOL [72]. The docking protocol for each protein was validated by superimposing the re-docked poses of co-crystallized ligands with the crystal conformations extracted from the PDB and the RMSD was calculated in PyMOL. The RMSD value should fall within a reliable range of 2 Å [M].

### 4.4. Molecular Dynamics Simulation

Molecular dynamics simulations were conducted to assess the binding stability, conformation, and interaction modes between the compounds (ursolic acid, α-amyrin, chrysin, and isoorientin) and receptors (1CET, 2BL9, and 4ZL4). The selected ligand–receptor complex files were subjected to molecular dynamics studies using GROMACS 2019.2 software [73]. The topologies of the selected ligands were obtained from the PRODRG server [74].

For the MD simulation, the complex structure was first minimized in a vacuum using the steepest descent algorithm for 5000 steps. The solvated complex was then placed in a cubic periodic box with dimensions of 0.5 nm, using a simple point charge (SPC) water model and maintained at a temperature of 310 K. The system was further equilibrated with an appropriate salt concentration of 0.15 M by adding Na^+^ and Cl^−^ ions. Each complex underwent a 50 ns simulation in the NPT ensemble (isothermal–isobaric, constant number of particles, pressure, and temperature).

Trajectory analysis, including root mean square deviation (RMSD), root mean square fluctuation (RMSF), radius of gyration (RG), and solvent accessible surface area (SASA), was performed using the GROMACS simulation package through the online server “WebGRO for Macromolecular Simulations” (https://simlab.uams.edu/, accessed on 5 January 2023). Graphs were generated using the Xmgrace tool of Grace Software [75] and Microsoft Excel.

### 4.5. Pharmacokinetic Properties

In silico pharmacokinetic and toxicity analyses of the compounds were conducted using ADMETlab 2.0 [57], a free online web server for analyzing ADMET properties. The isomeric SMILE codes of the compounds were entered into the server.

## 5. Conclusions

This study demonstrates the potential inhibitory activity of the four compounds identified in *C. obtusifolia* against important proteins of the Plasmodium genus (2BL9, 4ZL4, and 1CET). Specifically, α-amyrin and isoorientin exhibited the most promising activities and their pharmacokinetic behavior was predicted to be satisfactory, indicating their potential for administration without issues. Therefore, these two compounds possess the necessary characteristics, along with in vitro and in vivo studies, to be considered as potential antimalarial agents. Moreover, these results provide evidence supporting the traditional use of *C. obtusifolia* as a treatment for malaria.

## Figures and Tables

**Table 1:** Binding affinities and ligand–amino acid interactions.

Target	Ligand	Binding Affinities (Kcal/mol)	Amino Acid Interactions
2BL9	α-amyrin Chrysin Isoorientin Ursolic acid Pyrimithamine	−7.9 −8.7 −8.5 −7.8 −7.6	K55, Y56, V60, Y63, K169, Y192, S220, Y223, F232, C14, A15, D53, F57, S117, S120, I121, I173, G175, Y179 L39, L45, M54, F57, S116, S120, I121, Y125, I173, G175 K55, Y56, V60, Y63, Y192, S220, E221, F232 I13, C14, A15, L45, D53, M54, Y56, F57, I173, Y179
4ZL4	α-amyrin Chrysin Isoorientin Ursolic acid Wehi	−8.2 −9.6 −8.3 −7.8 −8.2	I24, D25, T285, F286, H288, K390, E399, L400, V402 Y27, D48, Y107, E109, L147, F148, V156 A28, G50, S51, C108, E109, D281, G283, T285, H288 D25, I24, E109, T285, F286, V354, K390 Y29, D48, S51, E109, Q151, G283, S284, T285, F286, H288, V354, V357
1CET	α-amyrin Chrysin Isoorientin Ursolic acid Chloroquine	−7.9 −7.8 −9.1 −7.7 −6.3	G11, G13, M14, P34, D35, I36, V37, A80, G81, F82, I105 G11, G13, D35, I36, A80, Y67, F82, G81, I105 S12, C13, I36, G11, N35, Y67, A80, G81, T83, E108 F34, N35, I36, V37, Y67, A80, G81, I105, I109 G11, F34, D35, A80, G81, F82, I36, Y67, I105

**Table 2:** Main pharmacokinetics parameters of compounds from *C. obtusifolia*.

Parameter	α-Amyrin	Chrysin	Isoorientin	Ursolic Acid
Formula	C_30_H_50_O	C_15_H_10_O_4_	C_21_H_20_O_11_	C_30_H_48_O_3_
MW (g/mol^−1^)	426.39	254.06	448.1	456.36
Lipinski	Accepted	Accepted	Rejected	Accepted
LogP (log mol/L)	7.603	3.58	0.708	6.453
HIA	0.003	0.012	0.909	0.004
Caco-2	−5.131	−4.874	−6.251	−5.396
PPB (%)	97.63	98.03	90.58	97.44
VD (L/kg)	1.251	0.493	0.834	0.672
BBB	0.056	0.02	0.011	0.265
CL (mL/min/kg)	5.609	5.131	4.067	3.538
T1/2	0.075	0.787	0.823	0.07
hERG	0.042	0.037	0.214	0.007
H-HT	0.156	0.079	0.133	0.435
Carcinogencity	0.012	0.317	0.037	0.085

## Data Availability

For data supporting the reported results, please contact the corresponding author.

## Supplementary Materials

The following supporting information can be downloaded at: https://www.mdpi.com/article/10.3390/molecules28196912/s1, Table S1: All pharmacokinetics results from ADMETlab 2.0.
